# The National Nosocomial Infections Surveillance in Iran. A 4 years report

**DOI:** 10.1186/1753-6561-5-S6-P243

**Published:** 2011-06-29

**Authors:** H Masoumi Asl

**Affiliations:** 1 Nosocomial Infection Department, Center for communicable disease control, Tehran, Iran, Islamic Republic Of

## Introduction / objectives

The national nosocomial infection surveillance program was established in Iran since 2007 based on National Nosocomial Infection Surveillance (NNIS) system definitions for four main groups of infections including pulmonary, urinary tract , blood stream and surgical site infections. This report is reflected a 4 year overview from 2007 to 2010.

## Methods

The nosocomial infection control department collected data from selected 100 hospitals with more than 200 beds, during a 4 year period and analyzed those using SPSS.16 software .

## Results

During the study period 6616520 patients were hospitalized in 100 hospitals. A total number of 57082 patients got nosocomial infection according to NNIS definitions. The infection rate in 100 hospitals from 2007 to 2010 were 0.6% , 0.87% , 0.96%, 1.1% respectively, (range 0.2% to 5.7%). Urinary tract infections (UTI) was the most common infection (28.9%) among reported cases, followed by pneumonia (PNEU, 28%), surgical site infections (SSI, 26.8%) and blood stream infections (BSI, 16.4%). The nosocomial infections in burn ward was more prevalent, followed by intensive care units, Hematology and Oncology, Pulmonary and kidney wards respectively. The overall mortality rate among patients affected by nosocomial infections during four years was 14.8%.

## Conclusion

The national nosocomial infection surveillance activities in Iran is a new program and the main weak point of the mentioned system is under-reporting which need educational interventions to change attitude of health workers and encourage them to detect , register and report nosocomial infection ,thus authorities would be able to make evidence-based decisions.

## Disclosure of interest

H. Masoumi Asl Other we have no conflicts of interest.

